# Radiomics and Prostate MRI: Current Role and Future Applications

**DOI:** 10.3390/jimaging7020034

**Published:** 2021-02-11

**Authors:** Giuseppe Cutaia, Giuseppe La Tona, Albert Comelli, Federica Vernuccio, Francesco Agnello, Cesare Gagliardo, Leonardo Salvaggio, Natale Quartuccio, Letterio Sturiale, Alessandro Stefano, Mauro Calamia, Gaspare Arnone, Massimo Midiri, Giuseppe Salvaggio

**Affiliations:** 1 Section of Radiology, BiND, University Hospital “Paolo Giaccone”, University of Palermo, Via del Vespro 129, 90127 Palermo, Italy; cutaiagiuseppe7@gmail.com (G.C.); giuseppe.latona@unipa.it (G.L.T.); federicavernuccio@gmail.com (F.V.); fra.agnello@libero.it (F.A.); cesare.gagliardo@unipa.it (C.G.); calamiamauro@gmail.com (M.C.); massimo.midiri@unipa.it (M.M.); p.salvaggio@libero.it (G.S.); 2 Ri.Med Foundation, Via Bandiera 11, 90133 Palermo, Italy; acomelli@fondazionerimed.com; 3 Nuclear Medicine Unit, ARNAS Ospedali Civico, Di Cristina e Benfratelli, 90133 Palermo, Italy; natale.quartuccio@arnascivico.it (N.Q.); letteriosturiale@gmail.com (L.S.); gasarno@interfree.it (G.A.); 4 Institute of Molecular Bioimaging and Physiology, National Research Council (IBFM-CNR), 90015 Cefalù, Italy; alessandro.stefano@ibfm.cnr.it

**Keywords:** prostate cancer, artificial intelligence, multiparametric magnetic resonance imaging, Gleason score, neoplasm recurrence, local

## Abstract

Multiparametric prostate magnetic resonance imaging (mpMRI) is widely used as a triage test for men at a risk of prostate cancer. However, the traditional role of mpMRI was confined to prostate cancer staging. Radiomics is the quantitative extraction and analysis of minable data from medical images; it is emerging as a promising tool to detect and categorize prostate lesions. In this paper we review the role of radiomics applied to prostate mpMRI in detection and localization of prostate cancer, prediction of Gleason score and PI-RADS classification, prediction of extracapsular extension and of biochemical recurrence. We also provide a future perspective of artificial intelligence (machine learning and deep learning) applied to the field of prostate cancer.

## 1. Introduction

Radiomics is a new frontier of medicine based on the extraction of quantitative features (named radiomic features) from radiological images that cannot be seen by radiologist’s naked eye and on the use of these data for the creation of clinical decision support systems. Radiomic features (such as intensity, shape, texture or wavelet) are extracted from medical images (CT, MRI or PET images) using advanced mathematical algorithms and providing valuable information for personalized therapy [1].

In the last decade, several studies highlighted the enormous potential of radiomics in both tumoral and nontumoral diseases of many organs and systems, including brain, lung, breast, gastrointestinal and genitourinary tracts.

Several studies have investigated the role of radiomics in neuro-oncology. The radiomics approach was used for the determination of WHO grades in patients with newly diagnosed gliomas [2,3] reaching an accuracy of approximately 90% and may thus be of clinical benefit in patients unsuitable for resection or biopsy. Furthermore, many studies [4,5,6] reported that radiomics and machine learning in conjunction with multiparametric MRI imaging in prediction of glioma local relapse after radiotherapy is the most promising approach, resulting in tumor infiltration map with an overall accuracy of approximately 90%. Several studies have also applied the radiomics approach to patients with neurodegenerative diseases, such as Alzheimer’s disease and Parkinson’s disease [7,8,9], and confirmed that radiomics analysis can reach comparable results to those obtained by using well-established MRI features when classifying neurological patients, adding complementary information about tissue heterogeneity. A recent clinical review [10] of radiomics application in lung cancer has shown a promising role of the radiomics approach in many fields, like diagnosis, distinguishing nonsmall cell lung cancer from other benign confounders or preinvasive conditions, and prediction of radiotherapy response and outcomes of patients. Concerning breast cancer, radiomics appears capable of offering imaging biomarkers, which are useful not only for diagnosing breast cancer, but also for predicting the treatment response and risk of recurrence [11]. For example, features extracted by MR images and MRI-based techniques are widely used to predict the response of neoadjuvant chemotherapy [12]. Furthermore, another study [13] showed that preoperative MRI signatures are able to estimate disease-free survival in patients with invasive breast cancer. Several studies used radiomic models and individual radiomic features to predict response to treatment in patients with gastrointestinal tumors (gastric and gastroesophageal cancer, colorectal cancer, hepatic cellular carcinoma and pancreatic cancer) [14], showing good predictive performance for response to treatment, despite using various strategies to construct predictive models. The radiomics approach was applied to bladder cancer and in kidney cancer evaluation, showing promising feasibility of radiomics for characterizing, staging and grading [15].

Many original papers have been published so far on the diagnostic and prognostic role of radiomics for prostate cancer. Therefore, the aim of this review is to analyze radiomic applications in the field of prostate MRI for prostate cancer. A particular point of interest is the analysis of the current and potential role of radiomics for tumor detection, prediction of prostate imaging-reporting archiving and data system (PI-RADS) and Gleason grading, and extracapsular extension of the tumor, as well as the assessment of tumor response and outcome.

## 2. Multiparametric MRI in Prostate Cancer

Multiparametric MRI (mpMRI) can be briefly summarized as a method of combining anatomic sequences (T1- and T2-weighted imaging) with functional sequences. The functional sequences of choice are diffusion-weighted imaging (DWI), including the calculation of apparent diffusion coefficient (ADC) and dynamic contrast-enhanced (DCE) maps [16].

While T1-weighted imaging is of limited use in assessing prostate morphology or in identifying a tumor within the gland, T2-weighted imaging provides high spatial resolution and defines the zonal anatomy by differentiating the peripheral zone from the transition zone [17]. Furthermore, in T2-weighted images, the peripheral zone has high signal intensity, while prostate cancer (PCa) appears as an area of lower signal. However, low T2 signal in the peripheral zone may also be seen in benign abnormalities, including prostatitis, fibrosis, scar tissue, postbiopsy hemorrhage or postirradiation. Introduction of mpMRI with the use of functional sequences may overcome these limitations; although the individual sequences are useful, T2-weighted imaging in combination with two functional sequences has been shown to provide better characterization of PCa [18,19,20].

In 2015, the prostate MR imaging study (PROMIS) group proposed a new diagnostic pathway in which mpMRI was used as a triage test for men at a risk of PCa [21]. The PROMIS study proposed mpMRI before prostate biopsy to avoid unnecessary biopsy, reduce false-negative results on targeted biopsy and increase the identification of clinically significant PCa not identified at digital rectal exploration or located in occult areas of the gland (apical distal, midline, subcapsular and anterior areas) [22,23].

For some years now, mpMRI has been considered an important diagnostic tool for the detection of PCa and it is recommended by the American College of Radiology and European Society of Urogenital Radiology (ESUR) [24,25].

Because prostate MRI interpretation can be subjective and inconsistent, suspicion scores for prostate cancer on an MRI (PI-RADS) have been developed on a 1- to 5-point scale (based on fixed criteria) for improved standardization of MRI interpretation and reporting. The first general version of the PI-RADS system was released in 2012 and included clinical guidelines for the performance of mpMRI along with a 5-point Likert scale for image interpretation [26]. A more refined scoring system were introduced in PI-RADS version 2, which was released in 2015 [27] and updated in 2019 with version 2.1, providing revised imaging acquisition parameters and a revised scoring system while maintaining the overall framework described in version 2 [28].

Most members of the PI-RADS Steering Committee recommend the use of 3T equipment for performing prostate MRI; even both 3T and 1.5T systems are acceptable [28]. Even if PI-RADS version 2.1 recommends the use endorectal coil with some 1.5T MRI systems, especially older ones, some studies demonstrated that prostate cancer foci may be detected by using a pelvic phase array receiver coil without reducing sensitivity as compared to an endorectal coil [29].

Following PI-RADS version 2.1 recommendations, MRI protocol for prostate study must include T1-weighted (T1W), T2-weighted (T2W), diffusion-weighted (DWI) and dynamic contrast enhanced (DCE) imaging [28]. PI-RADS version 2.1 confirms the dominant role of the DWI and T2W sequences in the peripheral zone (PZ) and in the transition zone (TZ), respectively [24].

A recent study comparing the accuracy of mpMRI in 29 studies revealed that the sensitivity and specificity of prebiopsy mpMRI ranged from 42 to 100% and from 12 to 100%, respectively [25]. Another recent study confirms that the sensitivity and the specificity of mpMRI for clinically significant PCa is 93% and 41%, respectively, with a negative predictive value and positive predictive value of 89% and 51%, respectively [30]. However, mpMRI has a low accuracy in the detection of small tumor foci, i.e., less than 0.5 cm^3^ [31].

## 3. Radiomics in Prostate Cancer

### 3.1. Detection and Localization of PCa

McNeal in 1988 divided the prostate gland into a peripheral zone (PZ), central zone and transitional zone (TZ), consisting of 70%, 25% and 5% of the prostate volume, respectively [32]. PCa arises in the PZ in 80% of cases; less commonly it originates in the TZ or, rarely, in the central zone. Some studies have found that tumors originating in the peripheral zone are the most aggressive [33].

T1W, T2W, DWI and DCE images provide important anatomical and functional information; however, about 25% of PCa in TZ may either not be recognized or mistaken for a benign prostatic hyperplasia nodule by mpMRI [34]

The use of computer-aided diagnosis tools used to complement radiologists’ assessments increases sensitivity and specificity in detecting PCa [35]. Recent radiomic publications for detection and location of PCa are summarized in Table 1.

Recently, there has been considerable interest in the role of texture features extracted from the computer (or radiomics) for detection [34]. In literature, there are many articles that aim to provide a radiomics approach based on mpMRI for the detection and localization of PCa in the peripheral and transition zones [36,37].

In 2016, Cameron et al. [38] proposed a quantitative comprehensive feature model called MAPS (morphology, asymmetry, physiology and size) based on radiomics for automatic detection of PCa and achieved an accuracy, sensitivity and specificity of 87%, 86% and 88%, respectively.

In 2018, Khalvati et al. [39] proposed an optimal multiscale radiomics-driven framework (MPCaD) for automated localization and detection of PCa. MPCaD leverages the full set of voxel-level quantitative radiomic features and incorporates region-level feature descriptors in a pipeline to better characterize and detect tumor regions. This framework incorporating computed high-b DWI (CHBDWI) and correlated diffusion imaging and was evaluated using clinical prostate mpMRI data from 30 patients. The authors demonstrated that the proposed framework exhibits enhanced differentiation of tumor and healthy tissue, reaching, with the full modalities model, a sensitivity, specificity and accuracy of 0.82, 0.89, and 0.86, respectively. The authors confirmed that the quantitative radiomic characteristics extracted from magnetic resonance imaging of the prostate can be used to detect and localize PCa.

Another study by Wibmer et al. [40] investigated whether Haralick texture analysis [41] of prostate MRI was useful for PCa detection on both the peripheral and transitional zone. They enrolled 147 patients extracted texture features (energy, entropy, correlation, homogeneity and inertia) from T2-weighted images and ADC maps. They found that in peripheral zone, on both T2-weighted images and ADC maps, entropy and inertia were significantly higher in prostate cancer areas than in noncancerous areas, whereas energy, correlation and homogeneity areas were significantly lower. For the transitional zone on ADC maps, entropy and inertia were significantly higher in the cancer areas than in noncancerous areas, whereas energy, correlation and homogeneity were significantly lower; on T2-weighted images, inertia was significantly higher in cancer lesions than in noncancerous areas whereas correlation was significantly lower. They concluded that Haralick-based texture features allowed for differentiating benign and malignant prostate tissue.

Nketiah et al. [42] performed a single arm, multicenter study to evaluate the potential of T2-weighted image-derived textural features for quantitative assessment of peripheral zone prostate cancer aggressiveness. They extracted traditional intensity histogram features from T2-weighted images and ADC maps and second- and high-order statistical image textural features based on gray level co-occurrence matrix (GLCM) and gray level run length matrix (GLRLM) from T2-weighted images. Spearman correlations were used to evaluate association between textural features and PCa grade groups. Mann–Whitney U-tests and support vector machine (SVM) classifier were evaluated to differentiate and classify low-(grade group 1) vs. intermediate/high-(grade group ≥ 2) aggressive cancers, respectively. The cross-validation scheme employed in support vector machine classifier training and testing across six institutional centers, and it works in the following way: at each iteration, data from one institution was held out for testing, and data from the remaining five institutions used for training. The mean classification accuracy across the centers was highest for the combined ADC and T2W intensity-textural features (84%) compared to ADC histogram (75%), T2W histogram (72%), T2W textural (72%) features alone or T2W histogram and texture (77%), T2W and ADC histogram (79%) combined.

### 3.2. Application of MR-Derived Metrics in PCa

The main application of mpMRI was localization and staging of PCa. Radiomics applied to mpMRI of the prostate have a wide application field, including tumor localization and detection, prediction of prognosis and follow-up after treatment. The great amount of data generation and the increasing volume of imaging data demanded the application of computerized methods to analyze mpMRI data and extract useful information.

Computerized quantitative analysis may lead to effective, accurate and reproducible analysis of large amounts of data from mpMRI. The main advantage of quantitative MRI will be elimination of subjective assessment by radiologists. A way to improve the characterization of focal lesions at multiparametric MR imaging could be to use computer-aided diagnosis (CAD) systems. CAD and artificial intelligence tools have been investigated for PCa diagnosis with mpMRI data. Several studies have been found CAD effective in aiding radiologists in PCa diagnosis. For example, Hambrock et al. [35] showed that the use of a CAD system in clinical condition could significantly improve the characterization of prostate lesions by less experienced readers. Niaf et al. [43] showed that a CAD system may improve the characterization of prostate lesions with mpMR imaging by increasing reading specificity. However, although numerous studies have shown promising results, the low specificity and high false-positive rate of CAD continue to be a major problem [44,45], so that further multicenter studies with large populations are needed for validation.

Another way to reduce the subjective evaluation by radiologists is the automatic segmentation method by the machine and deep learning approach. In the biomedical imaging field, target delineation is routinely used as the first step in any automated disease diagnosis system to obtain quantitative parameters from biomedical images. Deep learning algorithms have been applied in automatic segmentation of the prostate gland [46,47] with potential benefit for patient management personalization. As a future perspective, the integration of a deep learning network in radiological PACS would lead to a rapid and precise procedure of segmentation of the prostate gland, thus reducing interuser variability.

### 3.3. Prediction of Gleason Score and PI-RADS

Gleason score (GS) is the current clinical gold standard for prognostication of PCa [48]. GS allows for the stratification of patients into different risk groups based on architectural alterations of prostate tissue based on biopsy or prostatectomy [49], allowing discrimination between clinically significant and not significant PCa, defined as GS < 4 + 3, or as the maximum length of the tumor nucleus < 6 mm [50]. Some articles have confirmed that radiomics can predict GS in vivo [48]. Recent radiomic publications for prediction of GS are summarized in Table 2.

Many studies have been conducted in order to discriminate between clinically significant and not significant PCa using MRI radiomic features. Fehr et al. [51] in 2015 conducted a study on 147 patients and found that a combination of T2w and ADC magnetic resonance characteristics of the Haralick plot were able to distinguish low GS from intermediate and high GS with 92% accuracy. In another study, Cuocolo et al. [52] evaluated whether radiomic shape features derived from MR images could be effective in clinically significant PCa detection. They enrolled 75 patients and extracted ten shape features both from axial T2-weighted and ADC maps images, after lesion tridimensional segmentation. Using multivariable analysis, the parameter defined as surface area to volume ratio extracted from ADC maps was the strongest independent predictor of clinically significant PCa with AUC of 0.78, specificity of 97% and sensitivity of 56%.

Studies conducted to discriminate between clinically significant and not significant PCa using MRI radiomic features were performed using different MRI scanners (1.5T or 3T) and extracting radiomic features from T2-weighted and ADC maps [40,51,53,54,55]. These studies confirmed that radiomics can predict GS and allows differentiation between low to intermediate-high risk GS.

Therefore, in addition to confirming the presence of disease in vivo, radiomics is able to distinguish between clinically significant and not clinically significant PCa, allowing the early identification of patients who could be better candidates for active surveillance than definitive therapy [48].

The PI-RADS version 2.1, approved by the American College of Radiology, stratifies prostate lesions into different PCa risk categories [56]. However, this classification system has some limitations, including the potential interindividual variability in lesion categorization by radiologists [57]. Furthermore, at least 20–30% of the lesions indicated as PI-RADS 3 prove to be malignant lesions and this causes confusion in the diagnostic and therapeutic management of the patients [58].

Recently, some studies have verified the possibility of using radiomics to attribute PI-RADS scores. Wang et al. [57] used a machine learning-based system (support vector machine (SVM) based on radial basis function (RBF) kernel) to analyze radiomic features extracted from T2-weighted and DWI. They found that when radiomic features were added, the diagnostic performance of PI-RADS was improved with an increase in sensitivity from 79% to 94.4% in PCa in the peripheral zone, and from 73.4% to 91.6% in PCa in the transitional zone. The authors confirmed that MR radiomics can help to improve the performance of PI-RADS in clinically significant PCa.

In addition, Giambelluca et al. [58], using texture analysis software (MaZda 4.6), extracted first-, second- and third-order radiomic features from 46 PI-RADS 3 lesions segmented on T2-weighted and ADC maps. The authors found nine and six independent texture parameters on T2-weighted images and ADC maps, respectively, that significantly correlate with the final histological results. This confirmed that radiomic features can help to distinguish between PCa and nontumor tissue among PI_RADS 3 lesions.

Hou et al. [59] in 2020 developed a model integrating data extracted from T2W, DWI and ADC maps images of 271 patients. This model achieved promising performance in improving diagnostic accuracy in PI-RADS 3 by allowing clinically significant PCa to be differentiated from indolent and normal cases.

### 3.4. Prediction of Extracapsular Extension

Radical prostatectomy is considered the preferred approach for patients with localized PCa [60]. Accurate preoperative staging is important because any extracapsular extension (ECE) of the tumor influences the clinical decision-making process [61,62]. Various predictive models have been analyzed to try to understand the risk of ECE before surgery. The Partin tables and Memorial Sloan–Kettering (MSK) preradical prostatectomy nomograms are two of the most used models [63].

Many studies have shown that the accuracy of mpMRI for ECE detection varies from 62% to 76% but decreases up to 30% in the case of apex tumors [63].

Currently, neither predictive models nor mpMRI are effective in detecting the real risk of ECE due to their intrinsic limitations [63].

In recent years, a few studies proposed a radiomics approach to evaluate ECE. Ma et al. [63] proposed radiomic signatures based on T2W images to predict the side-specific probability of ECE for patients with PCa. They proposed a radiomics signature incorporating 17 selected features and demonstrated favorable discrimination capabilities in both the training and validation datasets, along with good calibration performances.

Losnegard et al. [64], in 2020, tested how the MRI radiomics approach performs in combination with preoperative clinical variables and radiology MRI interpretation. Texture features were extracted from T2-weighted and ADC maps using a Matlab toolbox (Lloyds function), and quantitative DCE time-series features were extracted using an in-house tool. Then, they used the extracted feature in a supervised machine learning setting in order to obtain the probabilities for patients to have ECE. A logistic regression model to obtain probabilities of ECE was calculated from clinical variables (PSA level, TNM stage, Gleason grade and score, percentage of positive core) and combined with radiomic features. They found that features extracted from T2-weighted and ADC maps were the best radiomic features while nonadditional benefit was added from DCE features. Moreover, they reached a good performance of combined models, with an AUC of 0.79 in prediction of ECE. They concluded that MR radiomics may represent a valuable adjunct to conventional prediction models for ECE.

The proposed radiomics signature had superior diagnostic performance compared, for example, to the visual assessments of radiologists, particularly for apical tumors [63].

In addition, Xu et al. [65] proposed a radiomics model based on mpMRI able to differentiate ECE and non-ECE lesions in preoperative time. They extracted radiomic features from T2-weighted images, DWI, ADC maps and DCE images using the Python package Pyradiomics (version 2.2.0). They retained 30 features by the use of a maximum relevancy and minimum redundancy algorithm. Finally, by the use of the least absolute shrinkage and selection operator (LASSO) regression algorithm, the final radiomics model was built and integrated with the clinical model to build a combined nomogram. They found that the combined nomogram outperformed the clinical model in diagnosing ECE, but was comparable with using the radiomics model alone.

A major limitation of all the reported studies, however, is that results are not validated on large population or in a multicenter study, and needs validation to have a role in future clinical application.

### 3.5. Prediction of Biochemical Recurrence after Treatment (Surgery or Radiotherapy)

Biochemical recurrence (BCR) occurs in 50% of patients who underwent radical prostatectomy, especially those with high-risk characteristics such as locally advanced disease (T3-4), positive margins (R1) or high Gleason score [66].

Adjuvant radiotherapy includes BCR-free survival, but its impact on overall survival is controversial and also a major cause of side effects [67].

For these reasons, radiotherapy (RT) is often provided exclusively in patients with BCR [67]. MpMRI allows recognition of seminal vesicle invasion or ECE, both of which are important predictors of biochemical relapse-free survival at five years [68].

To date, there are only a few studies on radiomics prediction of BCR. Bourbonne et al. [69] trained and validated a radiomics-based model that appears to be predictive of BCR and biochemical recurrence-free survival after radical prostatectomy in patients with PCa. They extracted first-order intensity metrics, second- and third-order textural features from T2-weighted and ADC maps of 107 patients. Then, a univariate and multivariate Cox regression analysis was performed to identify independent factors and the correlation with BCR was assessed. This model has a negative predictive value of 96% and could be used to identify patients at very low risk of recurrence. It would also help stratify patients after radical prostatectomy by helping the clinician adapt postoperative management. Using this model, patients with very low risk of BCR could avoid RT, thus reducing side effects, while patients with high risk of BCR could be offered intensified postsurgical monitoring or adjuvant RT.

Zhong et al. [70], in a retrospective study conducted on 91 patients, evaluated the relationship between pretreatment mpMRI radiomic features and BCR in patients with localized prostate cancer. The authors of this study validated a model with an improved predictive value over conventional imaging metrics that could facilitate MRI-based prognostic predictions and assist in the decision making for the individual treatment.

## 4. Future Perspective

Radiomics will play an important role in the future directions of personalized medicine. To achieve this goal and to allow the transition to clinical implementation, future radiomic studies should move to multimodality imaging (CT, MR, PET etc.) from current single modality imaging. The integration of image information of different scales from anatomical to molecular levels is mandatory.

For example, by combining information from mpMRI and prostate-specific membrane antigen (PSMA), PET might offer complementary information in PCa detection, overcoming the limitation of each single technique to identify the entire intraprostatic tumor amount. The aim of the study of Kesch et al. [71] was to define a genomic index lesion based on chromosomal copy number alterations (CNAs) as a marker for tumor aggressiveness in prostate biopsies in direct correlation to mpMRI and 68Ga-PSMA-PET/CT imaging features. A strong correspondence between the multi-imaging features and the genomic index lesions was identified. Consequently, this study demonstrates that multi-imaging features can guide to the genomically most aggressive region within the prostate. Papp et al. [72] aimed to investigate the diagnostic performance of PSMA PET/MRI in vivo models for predicting low vs. high lesion risk, together with BCR and overall patient risk, with machine learning. They demonstrated the potential to enhance risk classification in primary prostate cancer patients built on PET/MRI radiomics and machine learning without biopsy sampling.

In addition, to date, radiomics research is mostly retrospective studies performed in single institution, given the immediate availability of both imaging and clinical follow-up data. Conversely, there should be prospective studies performed in different institutions. On the other hand, it is obvious that radiomic features are vulnerable to imaging and reconstruction settings among different scanners and centers. For this reason, the integration of image-quality harmonization algorithms in radiomic workflows must be mandatory to correctly analyze data from different scales, obtaining more accurate and more robust results. In addition, the calculated features should be compliant with the ones provided in 2020 by the imaging biomarker standardization initiative (IBSI) standard [73] to set a robust methodological radiomics framework. Finally, not all extracted features carry out important information. Statistical models should be used to identify a subset of relevant features that correlate with the outcome in order to reduce the dimensionality of the problem, thus improving prediction accuracies [74].

Another major challenge concerning the radiomics is the theranostics. Theranostics combine specified therapeutics and specified diagnostics, such as through the use of radioactive iodine therapy. In this scenario, radio-labeled ligands targeting prostate-specific membrane antigen (PSMA) in PET imaging are expected to have good results in diagnosis and treatment of patients with hormone refractory prostate cancer [75]. Consequently, the usefulness of PET radiomic features for the aim of cancer marker evaluation, selection of patients expecting a better response, and development of prognostic markers is constantly growing [76]. Nevertheless, radiomics requires substantial amounts of data not easily available in the field of medical imaging, particularly in the nuclear medicine area

**Figure 1:**
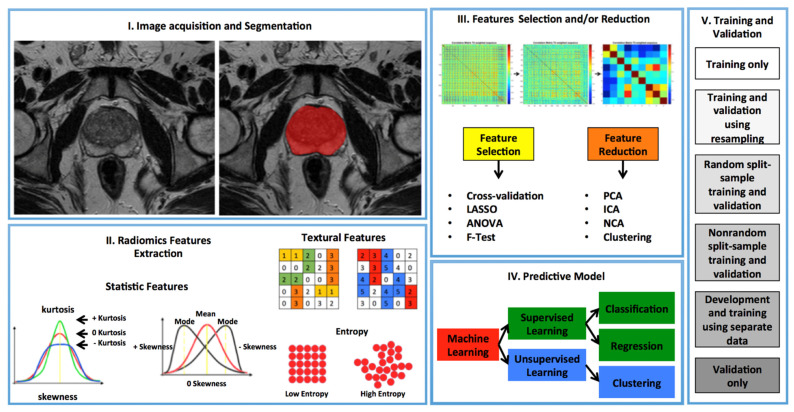
Radiomics workflow using artificial intelligence includes 5 steps: **I**. acquisition of radiological images and manual segmentation of the regions of interest (ROI); **II**. extraction of radiomics features from ROIs; **III**. methods of selection and/or reduction of the most significant radiomic features; **IV**. Artificial intelligence models for diagnosis prediction; **V**. Training and validation models.

As is well known, radiomics uses machine-learning or deep-learning techniques to build clinical models. In particular, deep learning methods automatically discover features from data using a general-purpose learning procedure with the advantage of removing a critical task from the current workflow of radiomics, i.e., the tumor segmentation (Figure 1). However, this requires more quantities of labeled data for the training process. For all these motivations, the scientific community should start a phase of a more systematic approach to radiomics, in such a way as to allow a real and immediate application in the clinical field.

## Figures and Tables

**Table 1:** Summary of radiomic manuscripts for detection and location of prostate cancer.

Author	MRI Sequences	Software and Features	Conclusion
Ginsburg SB at al., 2017	T2, ADC, DCE	Signal intensities on T2w and ADC values, kinetic features on DCE, edge descriptors, first-order statistical, co-occurrence, Gabor, Haar	Zone-aware classifier significantly improves the accuracy of cancer detection in the PZ
Bleker J et al., 2020	T2, ADC, DCE	Pyradiomics	Clinically significant PZ prostate cancer lesions can be quantified using a radiomics approach based on features extracted from T2w + DWI
Sidhu HS et al. 2017	T1, T2, ADC	TexRAD v.3.3	Textural evaluation technique may have particular relevance for such patients who are more likely to have TZ tumors that are systematically undersampled by TRUS
Cameron A et al., 2016	T2, DWI, ADC, Correlated Diffusion Imaging (CDI)	MAPS	In addition to being easier to interpret by radiologists, the MAPS feature model achieves higher classification performance (respect to conventional mpMRI)
Khalvati F et al., 2018	T2, DWI, Computed High-b Diffusion-Weighted Imaging (CHB-DWI), Correlated Diffusion Imaging (CDI), ADC	MPCAD	Quantitative radiomic features extracted from mpMRI of prostate can be utilized to detect and localize prostate cancer
Wibmer A et al., 2015	T2, ADC	Haralick Texture Analysis	Haralick-based texture features showed significant differences between noncancerous and malignant prostate tissue
Nketiah et al., 2021	T2, ADC	GLCM and GLRLM features, Spearman correlations, Mann–Whitney U-tests, SVM	T_2_W MRI-derived textural features correlated significantly with pathological findings (cancer grade group) from multiple institutions

**Table 2:** Summary of radiomic manuscripts for prediction of Gleason score.

Author	MRI Sequences	Software and Features	Conclusion
Fehr D et al., 2015	T2, ADC	In-house software implemented in Matlab (for first-order features); in-house software implemented in C++ (for Haralick features)	Addition of texture-based features drastically improves the classification accuracy of GS in comparison with using ADC mean or T2 mean alone
Cuocolo R et al., 2019	T2, ADC	Pyradiomics	Radiomics analysis through the quantitative assessment of geometric parameters has the potential to be used as a noninvasive test to predict GS for patients with clinically significant PCa
Wibmer A et al., 2015	T2, ADC	Haralick Texture Analysis	Haralick-based texture features showed significant differences in tumors with different GS
Chaddad A et al., 2018	T2, ADC	Gray level co-occurrence matrices (GLCMs), neighborhood gray-tone difference matrix (NGTDM), gray-level zone size matrix (GLSZM/GLZM)	Radiomics analysis has the potential to be used as a non-invasive test to predict GS
Min X et al., 2019	T2, DWI, ADC	In-house software implemented in Matlab (version 2014a)	mpMRI-based radiomics signature have the potential to noninvasively discriminate between clinically significant PCa and clinically insignificant PCa
Vignati A et al., 2015	T2, ADC	Gray level co-occurrence matrices (GLCMs)	Contrast and homogeneity GLCM features allow evaluation of PCa aggressiveness

## Data Availability

Not applicable.
